# Direct oral anticoagulants: patient reported adherence and minor bleedings

**DOI:** 10.1007/s11239-023-02797-8

**Published:** 2023-04-29

**Authors:** Amina Hayat, Anders Själander, Jonas Wallvik

**Affiliations:** 1 Internal Medicine Clinic, Sundsvall hospital, 856 43 Sundsvall, Sweden; 2 Department of Public Health and Clinical Medicine, Umeå University, Umeå, 981 87 Sweden

**Keywords:** Real world, Adherence, Minor bleedings, Atrial fibrillation, Direct oral anticoagulants, 8-item Morisky medication adherence scale

## Abstract

Data regarding adherence and minor bleeding on direct oral anticoagulants in everyday life are still sparse. Inclusion criteria: treatment initiated with dabigatran, rivaroxaban or apixaban in non-valvular atrial fibrillation patients from a center in northern Sweden between 2011 and 2019 (n = 668). Exclusion criteria: cognitive impairment, dose dispensing, need of interpreter or hospital admission (n = 67). By a telephone interview adherence was measured in 569 patients (response rate 94.8%) using the 8-item Morisky medication adherence scale and minor bleeding was asked for. CHA_2_DS_2_-VASc and HAS-BLED scores were collected from medical records. The number (n), mean age, mean treatment duration, mean (points) CHA_2_DS_2_-VASc and HAS-BLED scores was with dabigatran (n = 175, 73.3 years, 17.8 months, 3.6 p and 2.2 p), rivaroxaban (n = 198, 73.7 years, 21months, 3.8 p and 2.1 p) and apixaban (n = 196, 72.7 years, 15.2 months, 3.4 p and 2.1 p). Adherence was high for dabigatran, rivaroxaban and apixaban in 54%, 76% and 53%; intermediate in 37%, 20% and 37% or low in 9%, 4% and 10% respectively. High adherence (Morisky score 8) distinguished rivaroxaban (p < 0.0001) and in patients with CHA_2_DS_2_-VASc ≥ 4 p, (p < 0.0001). Patients on rivaroxaban/apixaban reported more minor bleedings (37% / 28%) compared to dabigatran (13%), (p < 0.001). Only 61% of the patients followed prescription. Adherence to rivaroxaban was significantly better, maybe due to the once daily dosing regimen, and furthermore among patients with higher risk for stroke. Minor bleedings were less common in the dabigatran group. The impact of minor bleedings on adherence and a possible relationship to clinical outcomes need to be further studied.

## Highlights

- Rivaroxaban showed significantly better adherence compared to dabigatran or apixaban including patients at higher risk for stroke (CHA2DS2-VASc score ≥ 4 p).
- Major bleeding on DOACs was 1,6%; self-reported minor bleedings was 17.8% per treatment 100 years.
- Self-reported minor bleedings were significantly less common in patients treated with dabigatran.
- Suboptimal adherence to DOACs merits further evaluation of impact on clinical efficacy.

## Introduction

Non-valvular atrial fibrillation (NVAF) is the most common cardiac arrhythmia worldwide [1] and has a prevalence of 3.1% in Sweden. Adherence to oral anticoagulation therapy (OAC) is fundamental as the therapy is effective in preventing stroke [2]. It is known that non-adherence result in poor clinical outcome by increase in morbidity and mortality [3].

The era of direct oral anticoagulants (DOACs) has provided patients with favorable drug characteristics by no requirement of routine monitoring or substantial food regulations as well as fixed dosing and fewer drug interactions [4]. The short half-life of DOACs is beneficial in the aspect of bleeding complications [4] but missed DOAC doses expose patients to greater risk of venous and arterial thromboembolism why concerns persist regarding adherence and lack of monitoring in patients treated with DOACs. Assessment of adherence can be measured using objective or subjective methods. The 8-item Morisky Medication Adherence Scale (MMSA-8) [5] is commonly used self-report screening tool and has been used in DOAC-treated patients to assess adherence [6–9]. It has been reported to correlate well with pharmacy refill rates [10] and has outstanding validation and reliability in chronic conditions [6].

The research regarding DOACs and bleeding complications in clinical practice and everyday life is still sparse. The principal objective was to study adherence and bleeding occurrence in a contemporary setting among patients with NVAF treated with DOACs registered in Auricula in the Sundsvall area, Sweden.

## Materials and methods

### Study population and patient baseline characteristics

We performed a study in everyday clinical setting to assess adherence and bleeding occurrence in patients with NVAF treated with DOACs in the Sundsvall area, Sweden. The Swedish national quality registry for AF and anticoagulation (Auricula) is a web-based facility giving support in dosing of Warfarin to the caregivers and as well as a registry for patients with AF on anticoagulants. Initially all patients with either dabigatran, rivaroxaban or apixaban between November 2011 and October 2016 were identified in Auricula. At that time the interviewed dabigatran cohort was small compared to rivaroxaban/apixaban (dabigatran n = 54, apixaban n = 114 and rivaroxaban n = 155) why the inclusion period was extended to July 2019 to enable statistical comparison with approximately 200 patients in each drug group. During the extended inclusion period patient lists was sorted according to age; youngest to eldest, and patients were selected from the patient list to allow similar mean age and equal gender ratio. Exclusion criteria prior to conducting the telephone interview were set dose dispensing, cognitive impairment, need of interpretation and current admission to a hospital. A total of 67 patients (10%) were excluded according to above mentioned criteria. Computerized medical records were reviewed in regard to obtain data on gender, age, treatment duration, indication for anticoagulation treatment, prescription fills, side effects and complications. CHA_2_DS_2_-VASc score was based on the presence of heart failure (ejection fraction ≤ 40%), hypertension, age 65–74 years, age ≥ 75 years, diabetes mellitus, prior thromboembolism, stroke or transient ischemic attack, vascular disease and gender category. These risk factors were assessed by using medical records. Included patients had a CHA_2_DS_2_‐VASc score ranging from 1 to 9 points.

### Assessment of adherence and complications

Adherence to DOAC treatment was assessed using a Swedish translated 8-item Morisky Medication Adherence Scale (MMSA-8) (Appendices). This questionnaire was originally used and validated in outpatient settings treated for hypertension [5]. It further received outstanding validation and reliability in patients with other chronic diseases [6]. The questionnaire consisted of total eight questions; the first seven items were dichotomous Yes/No responses while the last question had a 5-option response. The scale was used as a tool to adherence in DOAC treated patients by ranking adherence according to total score. Scores ranged from 0 to 8, where a score of 8 indicated high adherence, 6 or above but less than 8 indicated intermediate adherence and lower than a score of 6 was viewed as low adherence. On-treatment complications was set to major bleeding defined by International Society on Thrombosis and Haemostasis, minor bleeding defined as an overt bleeding event that does not fulfill the criteria of major bleeding, ischemic and hemorrhagic stroke, intracranial hemorrhage (ICH), myocardial infarction (MI) and venous thromboembolism (VTE).

### Telephone interview

The patients were posted written information; informed consent to participate in the study was obtained in written or oral form. The call was conducted as a part in a follow-up by the Anticoagulation Clinic in Sundsvall by a physician. Patients who did not attend the call were redialed a minimum of five times. In accordance to the telephone interview template (Appendices) each patient was given the chance to report possible bleeding complications and self-evaluate adherence by answering questions in accordance with the translated MMAS-8. As a part of the follow-up by the Anticoagulation Clinic patients were asked if they had knowledge about the requirement of renal function test when treated with DOACs.

### Statistical analysis

Categorical data were described by frequency, mean value, percentages and standard deviation. Chi square test with 95% or 99% confidence interval was used in the analysis of statistical significance.

## Results

### The cohort, inclusion and telephone interview

**Fig. 1:**
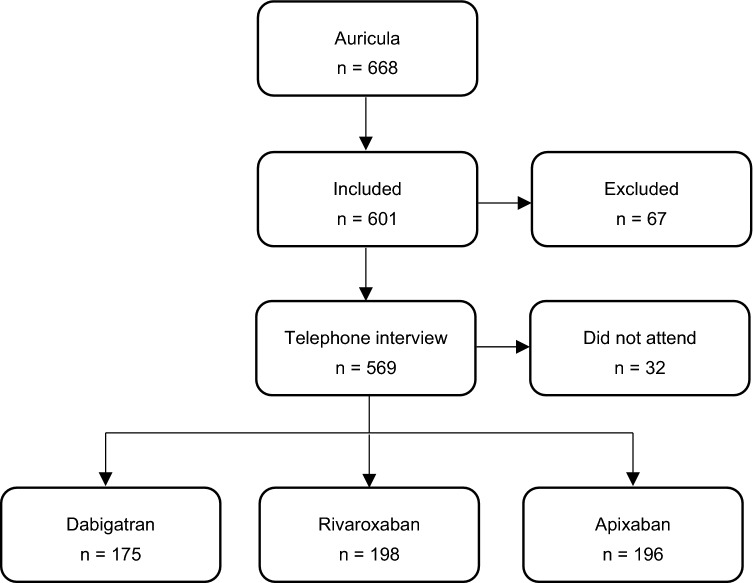
Flow chart of the study population. A total of 668 patients with non-valvular atrial fibrillation treated with dabigatran, apixaban or rivaroxaban were identified in Auricula, Sundsvall, the Swedish national quality registry for AF and anticoagulation. After exclusion of patients with dose dispensing, cognitive impairment, need of interpretation or current admission to a hospital; 601 patients remained. A telephone interview was conducted with 569 patients; additional 32 patients did not attend the call

A total of 668 patients with NVAF were informed of the study by letter and consented to participate. Prior to conducting the telephone interview 67 patients (10%) were excluded. A telephone interview was conducted with 569 patients; additional 32 patients (5%) did not attend the call (Fig. 1).

### Characteristics

Mean age was 73.2 years (SD 6.9 years), mean treatment duration 18 months (SD 13.4 months), mean CHA_2_DS_2_-VASc score 3.6 p (SD 1.5 p) and mean HAS-BLED score 2.2 p (SD 0.7 p) (Table 1). CHA_2_DS_2_-VASc score distribution in the entire cohort was ≥ 2 p (n = 537). The cohort was divided into groups of the specific prescribed DOAC, dabigatran (n = 175), rivaroxaban (n = 198) and apixaban n = 196). The proportion of patients on normal DOAC dosage was for dabigatran (74%), rivaroxaban (89%) and apixaban (80%); while reduced dose distribution was 26%, 11% and 20% respectively.

**Table 1:** Characteristics of NVAF patients treated with direct oral anticoagulation in Sundsvall, Sweden

	NOAC		Dabigatran		Rivaroxaban		Apixaban	
	n = 569		n = 175		n = 198		n = 196	
Women n (%)	272 (48)		83 (47)		95 (48)		94 (48)	
Reduced dose n (%)	108 (19)		46 (26)		22 (11)		40 (20)	
Mean age years (SD)	73.2	(6.9)	73.3	(5.3)	73.7	(6.7)	72.7	(8.3)
Treatment duration months (IQR1-IQR3)	19	(7–26)	20	(7–27)	20	(8–28)	15	(7–22)
Treatment years	847		259		340		248	
Mean HAS-BLED score (SD)	2.2	(0.7)	2.2	(0.7)	2.1	(0.7)	2.1	(0.7)
CHA_2_DS_2_-VASc score 1–3 n (%)	282	(50)	91	(52)	81	(41)	110	(56)
CHA_2_DS_2_-VASc score 4–9 n (%)	287	(50)	84	(48)	117	(59)	86	(44)

### Adherence in patients treated with DOAC

**Fig. 2:**
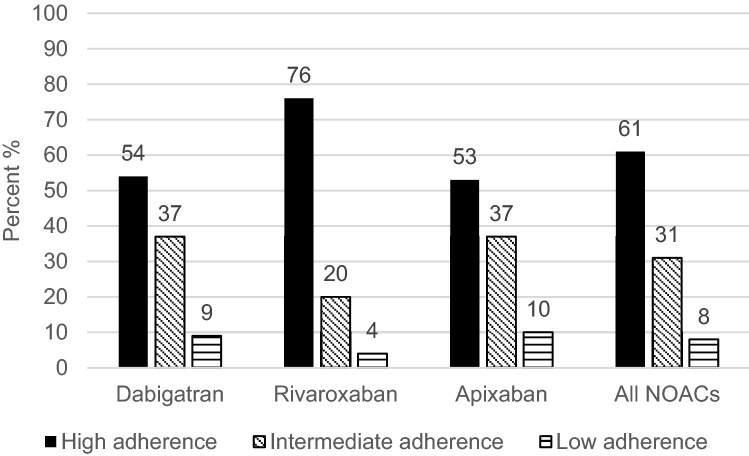
Adherence in DOAC treated NVAF-patients according to 8-item Morisky medication adherence scale. Dabigatran (n = 175), rivaroxaban (n = 198) and Apixaban (n = 196). Rivaroxaban had the highest share of patients (76%) with Morisky high adherence (score 8) compared to Morisky intermediate + low adherence (score < 8) to both dabigatran (54%) and apixaban (53%) (p < 0.0001)

In accordance with the MMAS-8 total score, degree of adherence to DOAC treatment was ranked either high, intermediate or low (Fig. 2). Morisky medication adherence was estimated to be high in 61% (n = 348) of the total 569 interviewed patients whereas Morisky intermediate and low adherence was 31% (n = 178) respectively 8% (n = 43) (Fig. 2). Rivaroxaban had the highest share of patients (76%, n = 150) with Morisky high adherence (score 8) (p < 0.0001) compared to Morisky intermediate + low adherence (score < 8) to both dabigatran (54%, n = 94) and apixaban (53%, n = 104) (Fig. 2). Score distribution showed that the majority of patients with Morisky intermediate adherence had a score of 7 or below.

### Characteristics of adherence assessed patients

**Fig. 3:**
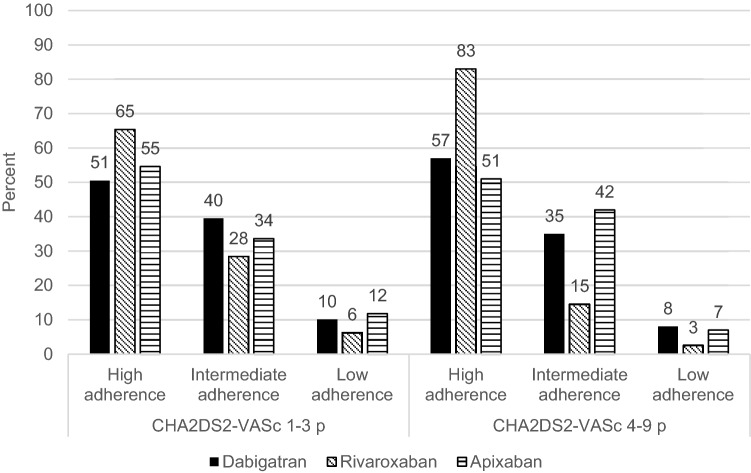
Relationship between CHA_2_DS_2_-VASc score and Morisky medication adherence assessed in NVAF patients with dabigatran (n = 175), rivaroxaban (n = 198) and apixaban (n = 196). High adherence (Morisky score 8) in patients with very high risk for stroke (CHA_2_DS_2_ VASc score ≥ 4 p) was significantly in favor of rivaroxaban compared to dabigatran (p < 0.0001) and apixaban (p < 0.00001) respectively

The number of patients assessed for degree of adherence treated with dabigatran, rivaroxaban or apixaban were 175, 198 and 196 respectively. Similarities were seen in mean age (73.3, 73.7 and 72.7 years), mean CHA_2_DS_2_-VASc score (3.6, 3.8 and 3.4 p) and HAS-BLED score (2.2, 2.1 and 2.1 p) while mean treatment duration (17.8, 21 and 15.2 months) differed among the studied DOACs (Table 1). High adherence (Morisky score 8) in patients with very high risk for stroke (CHA_2_DS_2_-VASc score ≥ 4 p) was significantly in favor of rivaroxaban compared to dabigatran (p < 0.001) and apixaban (p < 0.001) respectively (Fig. 3).

### Patient complications

**Fig. 4:**
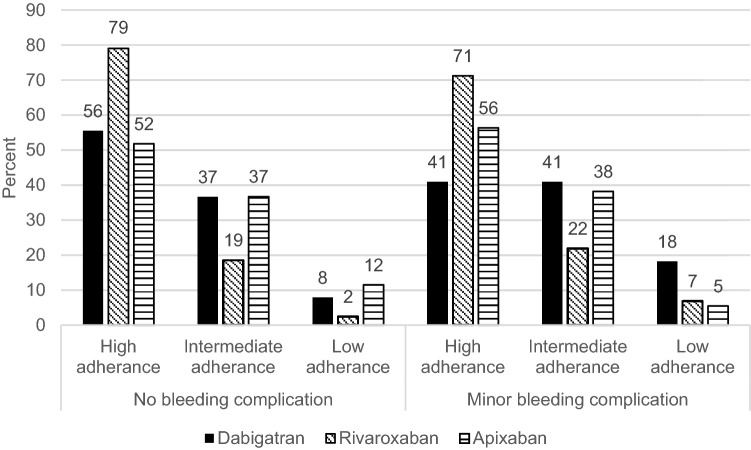
Relationship between occurrence of minor bleeding complication and Morisky medication adherence in non-valvular atrial fibrillation patients treated with dabigatran (no bleeding complication n = 153, minor bleeding complication n = 22, exclusion major bleeding n = 0), rivaroxaban (no bleeding complication n = 124, minor bleeding complication n = 73, exclusion major bleeding n = 1) or apixaban (no bleeding complication n = 139, minor bleeding complication n = 55, exclusion major bleeding n = 2). The occurrence of minor bleeding complication was not associated with lower degree of adherence in patients treating with dabigatran, rivaroxaban or apixaban

The total cases of ischemic stroke on DOAC treatment were 5 (0.9%) while on-treatment acute myocardial infarction was 6 (1%). A total of 9 (1.6%) patients suffered major bleeding of which 5 (0.9%) were major gastrointestinal (GI) bleeding. Side effects such as paresthesia, pruritus, urticarial, eczema, erythema, headache, dizziness, swelling, fatigue, nausea, diarrhea, obstipation, hypotension and dyspepsia were reported in 37 (6.5%) cases. Dabigatran (n = 22; 13%) had the lowest share of reported minor bleeding cases compared to both rivaroxaban (n = 73; 37%; p < 0.00001) and apixaban (n = 55; 28%; p < 0.001) (Table 2). Minor bleeding in patients treated with full respectively reduced dosage of dabigatran (full dosage n = 13; 10%, reduced dosage n = 9; 20%) was significantly (p < 0.05) lower compared to patients treated with full respectively reduced dosage of apixaban (full dosage n = 39; 25%, low dosage n = 16; 40%) or full dosage of rivaroxaban (full dosage n = 66; 38%) (Table 2). There was no significant difference in bleeding rate between rivaroxaban and apixaban (Table 2). The number and type of reported specific minor bleedings are seen in Table 3. Total minor bleeding events for dabigatran, rivaroxaban and apixaban was 31, 94 and 71 events respectively (Table 3). The occurrence of minor bleeding complications was not associated with lower degree of adherence for any of the studied DOACs (Fig. 4).

**Table 2:** Primary outcome in non-valvular atrial fibrillation patients treated with direct oral anticoagulants

			All bleeding n (%)	Major bleeding n (%)*	Major GI-bleeding n (%)	Minor bleeding n (%)	All bleeding annual bleeding rate	Major bleeding rate per 100 years	Minor bleeding rate per 100 years
Total	DOAC	n = 569	159 (28)	9 (1.6)	5 (0.9)	150 (26)	18.8	1.0	17.8
	Dabigatran	n = 175	24 (14)	2 (1.1)	1 (0.6)	22 (13)	9.3	0.8	8.5
	Rivaroxaban	n = 198	77 (39)	4 (2)	1 (0.5)	73 (37)	22.6	1.2	21.5
	Apixaban	n = 196	58 (30)	3 (1.5)	3 (1.5)	55 (28)	23.3	1.2	22.1
Full dosage	DOAC	n = 461	126 (27)	8 (1.7)	4 (0.9)	118 (26)	18.1	1.2	17.0
	Dabigatran	n = 129	15 (12)	2 (2)	1 (0.8)	13 (10)	7.6	1.0	6.6
	Rivaroxaban	n = 176	70 (40)	4 (2.3)	1 (0.6)	66 (38)	22.8	1.3	21.5
	Apixaban	n = 156	41 (26)	2 (1.3)	2 (1.3)	39 (25)	21.7	1.1	20.6
Reduced dosage	DOAC	n = 108	33 (31)	1 (0.9)	1 (0.9)	32 (30)	21.6	0.7	20.9
	Dabigatran	n = 46	9 (20)	0 (0)	0 (0)	9 (20)	14.5	0	14.5
	Rivaroxaban	n = 22	7 (32)	0 (0)	0 (0)	7 (32)	21.2	0	21.2
	Apixaban	n = 40	17 (43)	1 (2.5)	1 (2.5)	16 (40)	29.3	1.7	27.6

*Major bleeding as defined by International Society on Thrombosis and Haemostasis

**Table 3:** Reported minor bleedings in non-valvular atrial fibrillation patients treated with direct oral anticoagulants in Sundsvall. Sweden. Data presented as n (%)

	Dabigatran n = 175	Rivaroxaban n = 198	Apixaban n = 196
Total minor bleeding cases	22 (13)	73 (37)*	55 (28)*
Cumulative minor bleeding events			
0	147 (84)	125 (63)	141 (72)
1	16 (10)	55 (28)	39 (20)
2	5 (6)	15 (8)	16 (8)
3	0 (0)	3 (2)	0 (0)
4	0 (0)	0 (0)	0 (0)
5	1 (3)	0 (0)	0 (0)
Epitaxis	5 (3)	31 (16)	19 (10)
Hematoma	4 (2)	17 (9)	14 (7)
Rectal bleeding	6 (3)	16 (8)	15 (8)
Hematuria	6 (3)	9 (5)	6 (3)
Gingival bleeding	4 (2)	5 (3)	5 (3)
Eye bleeding	0 (0)	3 (2)	5 (3)
Wound bleeding	1 (1)	2 (1)	2 (1)
Mild hemoptysis	0 (0)	4 (2)	0 (0)
Muscle bleeding	0 (0)	2 (1)	0 (0)
Vaginal bleeding	3 (2)	1 (1)	3 (2)
Melena	2 (1)	2 (1)	2 (1)
Ear bleeding	0 (0)	1 (1)	0 (0)
Hemtospermia	0 (0)	1 (1)	0 (0)

*Major bleeding as defined by International Society on Thrombosis and Haemostasis

*Both rivaroxaban (37%) and apixaban (28%) had significantly (p < 0.00001 respectively p < 0.001) higher share of reported minor bleeding cases compared to dabigatran (13%)

## Discussion

In our Swedish real-world cohort treated with DOACs we found better adherence with rivaroxaban compared to both dabigatran and apixaban respectively. The difference persisted independently of CHA_2_DS_2_-VASc score. These findings are seen when adherence is categorized as either Morisky high adherence or Morisky intermediate + low adherence. Also, rivaroxaban showed significantly better Morisky high adherence in patients with higher risk for stroke compared to apixaban and dabigatran respectively. Though patients with cardiovascular disease and with perception of poor health are more likely to display better adherence [11] we on the contrary suggests that patients at higher risk for stroke taking apixaban or dabigatran might be exposed to consequences of suboptimal adherence. Additionally, our studygives insight to the variance in minor bleedings as we found significantly lower occurrence in the dabigatran group.

An attribute that might mark the inequality in adherence among the three studied DOACs could be dose regimen as rivaroxaban has once daily dosing in contrast to dabigatran and apixaban which have twice daily dosing. In DOAC treated patients medication persistence to a once daily intake regimen is significantly higher compared to a twice daily regimen [12], recently Gulpen et al. confirmed this using MMAS-8 with significantly better adherence to once daily dosing in rivaroxaban or endoxaban [13]. Previously Rossi et al. (mean CHA_2_DS_2_-VASc score 4.33 p) using the Morisky scale showed that twice daily dosage of DOACs being a main predictor of inadequate adherence 103 elderly subjects [14]. A study by McHorney et al. assessing real world medication adherence using proportion of days covered showed similarly to ours significantly favorable adherence rates for rivaroxaban compared to apixaban, dabigatran and warfarin. Moreover, patients treated with rivaroxaban in this study were less likely to discontinue therapy compared to above mentioned counterparts [15]. An American meta-analysis by Prentice et al. proportion of days covered for rivaroxaban was associated with increased adherence compared to dabigatran [16]. Besides difference in dose regimen there was no significant variance in mean values of age, treatment duration, CHA_2_DS_2_-VASc score and HAS-BLED in our study, challenging to distinguish other factors of influence on adherence.

Unlike findings in randomized controlled trials, our study of real-world use of DOACs did not demonstrate optimal adherence. This is consistent with previous studies, reporting more than 40% of patients with AF adhere suboptimally to OAC therapy [7, 10, 17]. Although our study has a larger cohort and used the MMAS-8 to assess adherence these number show similarities to our findings. However, Patel et al. compared adherence in patients with non-valvular AF with either VKA or DOACs using MMAS-8 noted no difference regardless of monitoring frequency [18].

Real life practice data concerning minor bleeding in DOAC treated patients mainly comes from large studies covering major bleedings, ischemic stroke and mortality. The increasing number of DOAC prescription raises the question of patient experience as prior work has shown impairment in quality of life due to minor bleedings [19]. Mitrovic et al. indicated that the major reason for discontinuation of DOAC therapy next to side effects is the occurrence of minor bleedings [20] and they have been shown to correlate with treatment duration and adherence [21]. Minor bleedings constitute an important issue in DOAC treated patients as the effect on adherence and the subsequent possible increment of stroke risk has barely been studied. Gulpen et al. recently proposed a one-year structured follow-up plan for DOAC treated patients which significantly improved the ratio of highly adherent patients compared to standard care [13]. This proposition seems valid as most of the DOAC discontinuation due to bleeding complications occur during the first year [20]. In a large study by Toorop et al., minor bleedings on DOACs were presented as a predictor for non-adherence [22]. Our study is one of few population-based studies to compare multiple DOAC medications and minor bleeding events. The occurrence of minor bleeding complications was not associated with lower degree of adherence for any of the studied DOACs. Despite high respectively similar adherence compared to its counterpart, patients treated with rivaroxaban or apixaban reported significantly more minor bleedings compared to dabigatran. This shows that prescription was followed in patients treated with rivaroxaban even though higher degree of minor bleedings. While apixaban having similar adherence and two dose regimen as dabigatran, it had significantly more minor bleedings. Our study could not conclude dose regimen as an independent risk factor for bleeding. Dabigatran is largely excreted by the kidneys compared to rivaroxaban and apixaban; therefore, prior assessment of renal function is of great importance, posing a risk for selection bias that may affect bleeding rates. The most common minor bleeding among patients treated with rivaroxaban or apixaban was nose bleeding while hematuria and rectal bleeding were most common among dabigatran treated patients. Interestingly Mitrovic et al. studying the same three DOACs and minor bleeding occurrence presented similar findings to ours. They could further deduce that type of DOAC, HAS-BLED score ≥ 3 p or previous VKA usage as potential risk factors for minor bleedings [23].

The main strength of the study is the statistical significance derived from a large cohort with has a response rate of nearly 95% on DOACs in everyday clinical practice. In addition, this study compares adherence and bleeding occurrence of three different DOACs and patients were included regardless of treatment duration or CHA_2_DS_2_-VASc score. The advantage of adherence assessment by telephone interview enables data to be collected in real-time, with less memory decay and actual behavior is obtained. Using an accepted self-report adherence measure the MMAS-8 allows adherence comparison with future studies. On the other hand, no conclusion could be made regarding the impact of reduced adherence on clinical outcome and complications. Beyond assessing prescription outtakes no objective assessment was made making our method susceptible to social desirability and recall bias.

In conclusion rates of non-adherence are high among patients on DOACs unlike findings in randomized controlled trials. Rivaroxaban was independently associated with adequate adherence and superior in patients at high risk for stroke. A lower incidence of minor bleedings was seen in patients treated with dabigatran. Further studies are needed to evaluate if minor bleedings lower adherence and have a consequential impact on stroke risk. Proposedly larger cohort studies with structured follow-up focusing on the frequency of minor bleedings and their relation to DOAC adherence would be the next step in this field.

## Abbreviations

NVAF: Non-valvular atrial fibrillation
DOACs: Direct oral anticoagulants
MMSA-8: 8-item Morisky medication adherence scale
ICH: Intracranial hemorrhage
MI: Myocardial infarction
VTE: Venous thromboembolism
GI: Gastrointestinal
